# Reform in Asylum Management

**Published:** 1894-12

**Authors:** 


### EDITORIAL DEPARTMENT.

F. E. DANIEL, M. D., Editor.
S. E. HUDSON, M. D., Managing Editor.
A. J. SMITH. M. D., Galveston, Associate Editor.

EDITORIAL STAFF:

PROF. J. E. THOMPSON, M. D., Texas Medical College, Galveston; Surgery.
PROF. WM. KEILLER, M. D., Texas Medical College, Galveston; Obstetrics and Gynecology.
PROF. DAVID CERNA, M. D., Texas Medical College, Galveston; Therapeutics.
PROF. A. J. SMITH, M. D., Texas Medical College, Galveston; Medicine.
DR. ISADORE DYER, Tulane University; Dermatology.
DR. R. H. L. BIBB, Saltillo, Mexico; Foreign Correspondent.

Official organ of the West Texas Medical Association, the Houston District Medical Association, the Austin District Medical Society, the Galveston County Medical Society, and several others

### REFORM IN ASYLUM MANAGEMENT.

The principle in physics that action and reaction are equal is exemplified in disease; depression and excitement, bearing the relation of cause and effect, have a direct ratio to each other. It is remarkable also that it is exemplified in many of the phases of social and industrial life and action. We see it strongly illustrated in fashions, for instance. It is the calm and the storm.

Our simile might be carried still further. We see the bound and rebound, the action and reaction, the depression and excitement, illustrated in labor strikes and political revolutions. Witness the great political upheaval in 1892, and the oscillation of the pendulum back to the other extreme in 1894. An evil is to be corrected. Indignation, resentment, zeal—what not, surely not deliberate reason, leads men to go to the other extreme, and hence in redressing one wrong, another is created.

The rebound, in the revolution that has taken place in the management and treatment of insane people, appears to have been excessive, and even beyond the depression. Time was, and not very long ago, when a "madman" was an object of fear and loathing, and a prison house was too good for him. He was possessed of a devil, and it had to be gotten out of him by stripes and blows, or if not to be dislodged, at least his satanship was to be prevented from doing his evil will, by straight jackets and chains.

Now, nothing is good enough for the insane. He is now recognized as a sick man, an irresponsible party, a ward of the State, and is an object of the tenderest solicitude. That is all very well; he should be nursed and taken care of; but the excess to which this solicitude has extended, borders on the absurd. To house a lot of idiots, imbeciles and insane, for the most part, of the pauper class, in palatial residences, and surround them with luxuries must strike any but the morbidly sentimental as ridiculous. For this purpose the State expends vast sums of money. Not only that, but the tender regard for the unfortunate extends to exempting him from work, even though he be physically able bodied. The folly of such policy is made the more manifest when it is reflected that such surroundings, and a constant state of idleness has been demonstrated to be detrimental to the patient. To that class who have delusions of grandeur, we should think it admirably calculated to heighten and maintain the delusions; while the unfortnnate dement would hardly be expected to recover his intellect by sitting day after day and gazing in idleness at white walls or masses of brick and mortar. It is enough to make one lose his mind.

Reform is called for: The time will soon arrive when the State cannot furnish magnificent asylum buildings for her insane, In fact it has arrived. Population is coming into Texas at the rate of over a million in a decade. We do not know the ratio of insane to population, but it may assumed that it is such that the insane would in a few years overflow a dozen such paleces as the State has erected and is maintaining at enormous cost at three points in Texas. In fact, the insane are now largely in excess of accommodation. The aggregate capacity of the three institutions is about fifteen hundred, and according to Superintendent White's report (see elsewhere in this issue), there are over one thousand insane now kept in prisons, and in private homes, cared for by friends. Let one ask himself, what will Texas do with her insane fifty years from now?

The reform demanded is not confined alone to quarters. More room is needed, and ungently needed, and comfort, convenience, space and durability should be the desiderata, and not elegance and grandeur. As pointed out by Dr. White, employment for the chronic insane,—for all who are capable of doing any kind of work, is urgently demanded. The therapeutic effect that congenial occupation would have upon these patients need hardly be dwelt upon; it readily suggests itself. But Dr. White appears to think that only agricultural pursuits are to be advised. There can be no doubt of the advisability of putting the able-bodied men to work in the way the doctor indicates; but the JOURNAL would suggest that there are many other pursuits which the inmates could follow, and should be instructed in. For instance, those of a purely mechanical nature,— the manufacture of brooms, buckets, and things of that kind, while the women could be profitably occupied in sewing, stitching shoes, book-binding and a thousand other little employments to which a large number of the afflicted are adapted.

Let the superintendent inaugurate some such reform as is here indicated, and we venture in his next report there would be good results to show. Let him classify his patieuts and assign each one to *some kind* of employment, and, if necessary, employ instructors. The blind pupils are taught the useful arts, and many are turned out, capable of making a living. It would not only be a source of revenue to the asylum, but the mind occupied, would sooner regain its equilibrium than if allowed to contemplate familiar objects day by day, and with nothing to do but brood over troubles, real or imaginary.

We give space to a part of Superintendent White's report, bearing upon the subject of reform, and commend it to the earnest consideration of our law makers.

As desirable as it is that some such reform should be inaugurated as above indicated; great as the improvement would be, in our judgment, the greatest evil of the present system is in the policy governing the appointment of the medical officers.

A superintendent and two resident assistant physicians are appointed with each incoming administration. Two years is hardly time to become acquainted with the inmates and their several ailments,—or to get the "hang" of the business,—and the manner of administering it, financially. It is not sufficient time to learn the individual characteristics and idiosyncrasies of the inmates,—for each case should be studied and treated independently of all others; there should be no routine treatment.

There is no position in which a physician can be placed that requires more tact and judgment, more nice discrimination, than here, in order to know and understand each case, and to put himself in sympathy with it. Confidence in, and respect for the medical officers must be secured from each patient. It is the

*sine qua non* of successful management. And when a superintendent has demonstrated a fitness and qualification for this work, when, by observation and active experience in the management of asylum affairs, and treatment of insane, he has acquired a training and a special aptitude for it, which can be acquired in no other way—the JOURNAL, in common, we believe, with the best sentiment of the medical profession, holds that it is detrimental to the interests of the State, and the welfare of the unfortunates for whom these great charities were founded, to remove him, and put in his place a new and untried and inexperienced man, whatever may be his learning, or his reputation. Worse still, when, as unfortunately it sometimes happens, his only claim is of a political nature, and the office is bestowed for partisan service.

We repeat, in redressing the great wrong done in the management and treatment of insane, we have gone to the opposite extreme; the pendulum has swung too far from the perpendicular, and an equilibrium should be established. Sentiment prompts the building of palaces and the maintenance of pauper insane in idleness, and politics works frequent changes in the management. Reason and common sense call for a more rational system, and demand that these offices be taken from the domain of politics.

